# Modeling and Mechanical Analysis of Materials and Structures in Civil Engineering

**DOI:** 10.3390/ma19204366

**Published:** 2026-10-17

**Authors:** Peng Wu, Aiguo Zhao

**Affiliations:** College of Civil Engineering, Nanjing Tech University, Nanjing 211816, China; shiang_37@163.com

## 1. Introduction

Modern civil engineering is increasingly shaped by simultaneous demands for structural safety, durability, sustainability, and computational efficiency. The continued development of laminated composites, smart and ultra-high-performance cementitious materials, timber products, lightweight steel systems, and other heterogeneous materials has expanded the range of mechanical phenomena that must be represented in analysis and design. Finite-element analysis remains a foundational numerical tool for structural and thermal problems [1]. In layered and multiphase systems, accurate prediction increasingly requires the combined treatment of through-thickness thermal fields, material heterogeneity, interfacial behavior, and time-dependent properties [2,3,4].

One prominent research direction is the transition from single-field and time-independent descriptions toward coupled and long-term analyses. Magnetoelectric laminates and cement-based piezoelectric smart composites illustrate the extension of structural materials toward coupled mechanical, electrical, and magnetic functionality [5,6]. Similar multi-physics challenges arise when environmental actions, temperature-dependent properties, moisture transport, or viscoelastic relaxation alter the mechanical response of layered materials. These developments highlight the need for models that preserve physical fidelity while remaining sufficiently efficient for parametric analysis and engineering application.

A second direction concerns the refinement of structural and constitutive models across different scales. Long-term serviceability, deterioration, connection behavior, interface mechanisms, material anisotropy, and tension–compression asymmetry can all modify the response predicted by conventional idealized models. Consequently, contemporary structural analysis increasingly combines analytical formulations, high-fidelity numerical simulations, and experimental validation to describe not only ultimate capacity but also service-life behavior and system-level performance.

Sustainability and data-driven prediction constitute a third major research trajectory. Recycled and high-performance cementitious materials introduce complex relationships among constituent properties, mixture proportions, microstructure, and macroscopic response. At the same time, machine-learning methods provide new opportunities for identifying nonlinear dependencies and supporting material design. Their engineering use, however, requires attention to dataset quality, generalization, uncertainty, and interpretability rather than predictive accuracy alone.

Against this background, this Special Issue, “Modeling and Mechanical Analysis of Materials and Structures in Civil Engineering”, collects eleven research articles spanning analytical solutions, experimentally validated finite-element models, multi-physics simulation, constitutive theory, sustainable material design, and data-driven prediction. Rather than viewing these studies as isolated applications, this Editorial organizes them into four connected themes: (i) multi-field coupling and time-dependent analysis of advanced materials; (ii) structural performance, durability, and system-level response; (iii) refined constitutive and micromechanical modeling; and (iv) sustainable materials and data-driven prediction. Together, the contributions show how contemporary civil engineering mechanics is evolving toward an integrated framework that combines physical modeling, numerical computation, experimental evidence, and intelligent prediction.

## 2. Overview of Contributions to This Special Issue

This Special Issue’s eleven papers address different materials, structural scales, and computational approaches, yet several common research directions can be identified. We emphasize scientific links among the contributions, highlighting how analytical mechanics, numerical simulation, experimental validation, and data-driven methods complement one another in current research on civil engineering materials and structures.

### 2.1. Multi-Field Coupling and Time-Dependent Analysis of Advanced Materials

The behavior of layered and multifunctional materials is often governed by interactions among mechanical response, environmental fields, interfaces, and time-dependent properties. Thermo-mechanical and -viscoelastic effects are especially important in laminated systems with temperature-sensitive constituents [3,4], whereas smart cement-based materials extend this concept to magneto- and piezoelectric interactions [5,6]. For timber systems, moisture-induced swelling and shrinkage and their associated stiffness changes are central to mechanical response [7,8]. Meanwhile, in laminated glass, time- and temperature-dependent polymer interlayer behavior requires viscoelastic descriptions of composite action [9,10]. These characteristics motivate solution strategies that combine three-dimensional mechanics with heat or moisture transport, state-space formulations, transfer matrices, multi-physics finite-element environments, and transform-domain methods.

Pan et al. (Contribution 1) developed an exact three-dimensional thermoelastic model for the bending response of fiber metal laminate plates subjected to combined thermal and mechanical loading, incorporating orthotropy and temperature-dependent properties with heat-conduction theory, Fourier-series expansion, the state-space approach, and the transfer-matrix method. The analytical solution agreed closely with finite-element results, with errors generally below 2% away from the outer surfaces, and also showed satisfactory agreement with experimental results in the linear range. A particularly important finding was that the conventional superposition principle can become inaccurate when temperature-induced modulus degradation is significant, with errors reaching 30.39%. To address this limitation, the authors introduced a modified superposition principle accounting for property degradation. Their parametric analysis further demonstrated the combined influence of temperature distribution and lamina number on stresses and displacements.

Huang et al. (Contribution 3) investigated 2–2 cement-based magnetoelectric composites using a coupled COMSOL Multiphysics 6.2 model. Their numerical framework reproduces stress–strain transfer, displacement, polarization, and voltage evolution in the magnetostrictive and piezoelectric phases. Their results show pronounced non-uniformity in the mechanical fields due to geometry and boundary constraints, while the output voltage increases with the bias magnetic field before approaching saturation. Under alternating magnetic excitation, resonance behavior is governed jointly by the excitation conditions and structural dimensions, and the study also demonstrates that the thickness ratio of the functional phases has a strong and non-uniform influence on strain transfer and magnetoelectric conversion efficiency. These findings provide a modeling basis for the design of multifunctional cement-based sensing and energy-conversion materials.

Xu et al. (Contribution 6) addressed the coupled effect of mechanical loading and moisture variation in cross-laminated timber (CLT) plates. A three-dimensional analytical framework was established by combining moisture diffusion, moisture-induced deformation, moisture-dependent elastic properties, the state-space method, and transfer-matrix relations through the full thickness of the laminated materials. Verification against refined finite-element simulations showed their solution provided high computational efficiency. The study showed that the traditional superposition principle is not generally valid when mechanical properties vary with moisture content, with errors reaching 18.2% in the investigated cases, and a modified superposition principle was therefore proposed. The analysis further clarified the interaction between externally induced and moisture-gradient-induced bending, as well as the roles of response symmetry, wood species, and anisotropic swelling behavior.

Zhu et al. (Contribution 11) extended the treatment of layered structures to the long-term bending of laminated glass plates with temperature-dependent viscoelastic interlayers. Glass plies were modeled with three-dimensional elasticity and the state-space method, while the polymer interlayer was represented using a generalized Maxwell model together with the Williams–Landel–Ferry relation. Boltzmann superposition, double Fourier series, and the Laplace transform were used to obtain the analytical response. The solution agreed with published long-term experiments and finite-element simulations, with average experimental errors below 4% and finite-element discrepancies of about 1.5% for the benchmark cases. For a representative case, the analytical formulation was approximately 1.13 × 10^3^ times faster than the finite-element simulation and required only about 10.88% of its memory. The results further show that temperature mainly changes relaxation rate, whereas interlayer material, thickness, number of glass layers, and aspect ratio govern long-term composite action and deformation.

### 2.2. Structural Performance, Durability, and System-Level Response

At the structural scale, accurate prediction requires more than a detailed material model. Prestressed concrete structures are particularly sensitive to long-term prestress loss, creep, and shrinkage [11,12], while cold-formed steel wall systems depend strongly on the interaction among framing, sheathing, and connections [13,14]. Concrete pavement joints provide another example in which load transfer is governed by interface condition, crack geometry, and aggregate interlock [15,16]. These issues show why service-life effects and system-level interaction must be incorporated into practical structural assessment.

Won et al. (Contribution 2) developed an experimentally calibrated ABAQUS/Explicit model for prestressed concrete girders subjected to long-term prestress loss and deterioration, reproducing load–deflection behavior, crack initiation, stiffness transition, maximum load, and post-peak ductile response for post-tensioned girders with different tendon strengths. Parametric studies then examined residual prestress, grouting defects, concrete-strength reduction, and tendon cross-sectional loss. The results indicate that prestress loss primarily affects the serviceability limit state by reducing stiffness, increasing deflection, and promoting earlier cracking, whereas ultimate capacity is more sensitive to the deterioration of material properties and cross-sectional area. The coexistence of multiple degradation mechanisms can accelerate performance loss, underscoring the importance of life-cycle-oriented assessment and maintenance planning.

Kang et al. (Contribution 5) investigated the lateral resistance of a modular cold-formed steel shear wall connected by rectangular steel tubes and quantified its contribution to multi-story frame response. Four full-scale specimens were used to validate detailed and simplified numerical models, with their simplified spring-based representation capturing principal hysteretic and deformation characteristics while reducing computational demand, making it suitable for system-level analysis. Simulations of six-story frames showed that the modular walls reduced roof lateral displacement by 11–71% and maximum inter-story drift ratios by 15–67% under the considered seismic actions. This work is significant because it links component testing with an efficient modeling strategy for structural-system analysis, thereby facilitating the practical incorporation of modular lightweight walls into frame design.

Jamieson and White (Contribution 10) focused on load transfer across contraction joints in rigid aircraft pavements. Instead of representing aggregate interlock solely with an equivalent spring, they characterized joint interfaces using an idealized two-dimensional sinusoidal crack geometry and evaluated the effects of joint opening, wheel configuration, sub-base type, crack shape, saw-cut depth, and subgrade strength through finite-element and factorial analyses. Joint opening was identified as the dominant variable, and the investigated load-transfer response approached zero for openings between approximately 2 and 3 mm. A cubic relationship between load transfer and displacement-based load-transfer efficiency was also proposed. Although the model is theoretical and requires further validation using field data and concrete cores, the study demonstrates the value of representing physical crack morphology more explicitly in pavement-joint analysis.

### 2.3. Refined Constitutive and Micromechanical Modeling

Constitutive modeling remains central to the predictive capability of structural analysis. Classical formulations often assume equal tensile and compressive properties or isotropic material response, assumptions that may be inadequate for bimodular solids and granular media. Tension–compression asymmetry can modify yielding, plastic-zone development, and residual deformation in bending [17,18], whereas the macroscopic anisotropy of sand is closely linked to particle arrangement, fabric evolution, and contact-force transmission [19,20]. Two contributions in this Special Issue address these issues by introducing physically interpretable refinements to conventional constitutive descriptions.

He et al. (Contribution 7) revisited the classical elastic–plastic cantilever-beam problem for materials with different elastic moduli and yield strengths in tension and compression. Closed-form relations were developed for the loading and unloading stages, including plastic-zone development, ultimate free-end deflection, and residual deflection after unloading, and the theoretical results were verified numerically. The analysis shows that tension–compression asymmetry enlarges the elastic–plastic deformation response and changes the conventional evolution of the plastic region—once a plastic hinge forms, the plastic zone can exceed the classical one-third beam-length result, and both the ultimate and residual deflections increase as the asymmetry becomes more pronounced. These results highlight the need to account for bimodular and strength-differential effects when refined deformation or limit-state assessments are required.

Yu et al. (Contribution 9) developed a micromechanics-based elastoplastic model for anisotropic sand by introducing a true stress tensor that couples the tensors for macroscopic stress and fabric evolution. Mapping anisotropic yielding and deformation into an equivalent true-stress space, this proposed tensor was combined with critical-state theory, fabric evolution, an energy-based stress-dilatancy relation, and coordination-number evolution. The model parameters retain physical meaning and can be calibrated using standard laboratory tests. Validation against conventional triaxial experiments, true-triaxial simulations, and data for inherently anisotropic Toyoura sand showed that the framework can reproduce stress–strain response, volumetric deformation, loading-direction effects, and behavior under complex stress paths. The work therefore provides a mechanistically motivated bridge between granular microstructure and macroscopic anisotropic constitutive response.

### 2.4. Sustainable Materials and Data-Driven Prediction

The increasing availability of experimental databases has created new possibilities for combining sustainable material development with data-driven modeling. As recycled aggregate concrete (RAC) is intrinsically heterogeneous, both its environmental assessment and mechanical-property prediction are affected by the variability of its recycled constituents [21,22]. Ultra-high-performance concrete (UHPC) presents a different but related challenge because its response depends on strongly coupled mixture variables, particle packing, interfacial characteristics, and fiber-bridging mechanisms [23,24]. Machine-learning approaches have already been used to predict the elastic and mechanical properties of heterogeneous cementitious materials [25,26], and while more recent tabular foundation models can improve prediction in small-data settings [27], dataset representation bias remains an important constraint on model generalizability [28].

Lu et al. (Contribution 4) proposed a framework for trustworthy prediction of RAC elastic modulus by integrating the Tabular Prior-data Fitted Network (TabPFN) with Monte Carlo Bias Estimation. A database of 1161 specimens was assembled, and the TabPFN model achieved an R^2^ of 0.912 and a root mean square error (RMSE) of 1.65 GPa without task-specific hyperparameter tuning, outperforming the benchmark algorithms considered in the study. Monte Carlo bias analysis identified underrepresented regions, particularly for high-strength RAC, and was used to guide targeted data augmentation. SHapley Additive exPlanations (SHAP) analysis indicated that compressive strength is the dominant predictor while retaining physically meaningful effects from recycled aggregate replacement and water absorption. The study is notable because it treats prediction accuracy, dataset bias, generalization, and interpretability as connected requirements rather than isolated objectives.

Wang et al. (Contribution 8) combined orthogonal experiments, microstructural characterization, and artificial neural network (ANN) prediction to investigate coarse-aggregate UHPC reinforced with hybrid fibers. The hybrid steel/composite-fiber system increased compressive and flexural strength by 12.4% and 14% relative to the comparison system, respectively, while scanning electron microscopy and X-ray computed tomography linked the improved ductility to a denser interfacial transition zone and multi-scale crack-bridging network. The ANN captured nonlinear interactions among mixture variables, reaching an R^2^ of 0.9703 and RMSE of 2.11 MPa for compressive-strength prediction. The study further demonstrated that fly ash and fiber parameters should be co-optimized rather than treated independently. By connecting macroscopic testing, microstructural evidence, and data-driven prediction, this contribution illustrates a broader trend toward integrated material design rather than purely empirical mixture optimization.

Taken together, the eleven contributions illustrate a progressive shift from isolated modeling techniques toward integrated multi-scale and -method research frameworks. Exact and semi-analytical solutions remain valuable for revealing governing mechanisms and providing computationally efficient benchmarks; finite-element models enable complex geometries, deterioration states, and structural-system simulations; experiments remain indispensable for calibration and validation; and machine-learning methods provide new tools for extracting nonlinear relationships from heterogeneous datasets. The central challenge is therefore not replacing one class of methods with another, but combining them in a manner that preserves physical consistency, transparency, and engineering relevance.

## 3. Conclusions and Outlook

This Special Issue demonstrates the breadth of current research on the modeling and mechanical analysis of civil engineering materials and structures. Its contributions range from exact three-dimensional solutions for layered plates to experimentally validated structural simulations, multi-physics modeling of smart cement-based composites, micromechanics-based constitutive theory, and artificial-intelligence-assisted prediction. Across these different topics, a common objective links the contributions: representing material and structural behavior more realistically while maintaining sufficient efficiency for parametric studies, design-oriented analysis, and engineering decision-making.

Several research trends emerge. First, multi-field and time-dependent analysis will become increasingly important as advanced materials are subjected to coupled thermal, moisture, electromagnetic, and sustained-loading environments. Second, constitutive and interface models are expected to incorporate more direct links among microstructure, anisotropy, damage, degradation, and macroscopic response. Third, structural assessment should move further toward life-cycle-oriented frameworks that consider serviceability, durability, environmental sensitivity, and deterioration together with ultimate capacity. Finally, data-driven approaches should increasingly be combined with physical constraints, uncertainty quantification, bias assessment, and interpretable variables so that predictive performance is accompanied by engineering trustworthiness.

Future progress will therefore depend on the coordinated use of analytical mechanics, high-fidelity numerical simulation, advanced experimental characterization, monitoring data, and physics-guided or hybrid machine-learning methods. Benchmark problems and transparent validation procedures will remain essential for evaluating transferability across materials, geometries, and loading conditions. We hope that the contributions to this Special Issue will encourage further collaboration across mechanics, material science, structural engineering, and computational intelligence and support the development of more durable, resource-efficient, safe, and intelligent civil engineering systems.
